# Silent Hypoxia From Benzocaine-Induced Methemoglobinemia Following Transesophageal Echocardiogram

**DOI:** 10.7759/cureus.87109

**Published:** 2025-07-01

**Authors:** Benjamin Easow, Sandhra Jiby, Tijin Mathew, Lydia George, Kevin Meek

**Affiliations:** 1 Internal Medicine, Southeast Health Medical Center, Dothan, USA; 2 Internal Medicine, Dr. Somervell Memorial CSI Medical College, Thiruvananthapuram, IND

**Keywords:** benzocaine toxicity, cyanosis, methemoglobinemia, methylene blue, mrsa bacteremia, septic pulmonary emboli, silent hypoxia, topical anesthetic complication, transesophageal echocardiogram

## Abstract

Methemoglobinemia is a rare but potentially life-threatening cause of hypoxia, often presenting with cyanosis and low oxygen saturation unresponsive to supplemental oxygen. We report the case of a 32-year-old woman with a complex medical history including morbid obesity, asthma, type 2 diabetes mellitus, and hypertension, who was admitted with MRSA bacteremia and septic pulmonary emboli. After a nondiagnostic transthoracic echocardiogram, she underwent a transesophageal echocardiogram (TEE), during which topical benzocaine spray was administered for oro-esophageal anesthesia. Following the procedure, she developed asymptomatic but profound hypoxemia, requiring rapid escalation from room air to 100% oxygen via non-rebreather mask, despite denying dyspnea. Arterial blood gas analysis revealed a methemoglobin level of 31.8%, confirming benzocaine-induced methemoglobinemia. She was promptly treated with methylene blue, resulting in rapid clinical improvement and return to room air the next morning. This case underscores the importance of recognizing silent hypoxia following TEE, particularly when benzocaine-containing anesthetics are used. Early recognition and treatment are essential to prevent unnecessary escalation of care and potential morbidity.

## Introduction

Methemoglobinemia is a rare, potentially life-threatening condition in which hemoglobin is oxidized to methemoglobin (MetHb), rendering it incapable of binding and delivering oxygen effectively to tissues. Although normally present in trace amounts (<1%), MetHb levels can increase significantly following exposure to certain oxidizing agents, including topical anesthetics like benzocaine [1]. It can be congenital or acquired, with the latter more frequently resulting from medication or chemical exposure [2,3]. Benzocaine is one of the most commonly implicated agents, particularly during procedures such as bronchoscopy, endoscopy, and transesophageal echocardiography (TEE) [4,5].

In this case, TEE was indicated to evaluate for possible infective endocarditis in the setting of methicillin-resistant *Staphylococcus aureus *(MRSA) bacteremia and septic emboli, as the initial transthoracic echocardiogram (TTE) was nondiagnostic. Shortly after the procedure, the patient developed unexpected hypoxemia without clinical distress. The diagnosis of methemoglobinemia was suspected based on a discrepancy between low oxygen saturation on pulse oximetry and relatively preserved respiratory effort and physical findings. This highlights the importance of clinical awareness in detecting silent hypoxia following benzocaine exposure.

## Case presentation

A 32-year-old woman with a complex medical history, including morbid obesity (BMI 51), asthma, type 2 diabetes mellitus, and hypertension, presented to the emergency department with three days of fever, shortness of breath, and a draining gluteal abscess. Physical examination was notable for the abscess but was otherwise unremarkable. Initial laboratory workup revealed leukocytosis and stable renal function (Table 1). Blood cultures subsequently grew MRSA. A contrast-enhanced chest CT revealed multiple scattered peripheral nodular opacities consistent with septic emboli.

**Table 1:** Initial Laboratory Results (Day of Admission)

Test	Patient Result	Reference Range
White blood cell count (WBC)	14,000 /µL	4,000–11,000 /µL
Hemoglobin (Hb)	11.2 g/dL	12–16 g/dL
Platelets (PLT)	310,000 /µL	150,000–400,000 /µL
Creatinine	1.1 mg/dL	0.6–1.3 mg/dL
Blood urea nitrogen (BUN)	18 mg/dL	7–20 mg/dL

The patient was empirically started on intravenous vancomycin, cefepime, and metronidazole to provide broad coverage, including gram-negative and anaerobic organisms, given her comorbidities and presumed source of infection. Once blood cultures confirmed MRSA, antibiotic therapy was narrowed to vancomycin monotherapy. The abscess was surgically debrided. 

To evaluate for infective endocarditis, a transthoracic echocardiogram (TTE) was performed but was nondiagnostic. A transesophageal echocardiogram (TEE) was subsequently performed using topical benzocaine (HurriCaine spray) for local oropharyngeal anesthesia. The TEE revealed no valvular vegetations or intracardiac pathology. Unfortunately, TTE and TEE images were not available for inclusion, as institutional policy typically archives echocardiographic imaging only when significant abnormalities are detected.

Within 30-45 minutes of the TEE, the patient developed asymptomatic hypoxemia, with oxygen saturations dropping into the mid-80s on room air. She denied dyspnea or chest pain and appeared clinically stable. Oxygen therapy was escalated from 2 L/min via nasal cannula to 6 L/min, and subsequently to 15 L/min via a non-rebreather mask over the next 30 minutes, but her oxygen saturation remained persistently low on pulse oximetry (SpO₂ 85%). Physical examination remained unremarkable, and her lungs were clear to auscultation. A saturation gap was noted, with pulse oximetry showing 85%, while arterial blood gas with co-oximetry revealed a SaO₂ of 65.3% and a methemoglobin level of 31.8% (reference: <1.5%) (Table 2). Although a baseline MetHb level was not available prior to the procedure, the rapid onset and known benzocaine exposure strongly supported an acquired etiology. Alternative causes of hypoxia, including pulmonary embolism, pneumothorax, and device malfunction, were considered and excluded based on imaging and clinical findings.

**Table 2:** Co-oximetry (Post-TEE) All values were measured via arterial blood gas with co-oximetry following transesophageal echocardiography. Reference ranges are institution-specific and may vary. TEE: transesophageal echocardiogram.

Test	Patient Result	Reference Range
Carboxyhemoglobin (COHb)	0.0%	<1.5%
Methemoglobin (MetHb)	31.8%	<1.5%
Oxyhemoglobin (O₂Hb)	65.3%	>95%
Calculated oxygen content	11.5 mL/dL	15–22 mL/dL (approximate)
Total hemoglobin (tHb)	12.7 g/dL	12–16 g/dL (female)

She was treated with 100 mg (approximately 1.5 mg/kg) of intravenous methylene blue in the preoperative holding area, with the anesthesia team present and in coordination with the ICU team. A second dose of 1 mg/kg was ordered but was held due to rapid clinical improvement. G6PD deficiency was not formally excluded prior to treatment; however, given the urgency and low clinical suspicion, methylene blue was administered under close hemodynamic and oxygenation monitoring. The patient was weaned to room air within 12 hours and had fully recovered by the following morning.

## Discussion

Methemoglobinemia results from the oxidation of the ferrous (Fe²⁺) iron in hemoglobin to the ferric (Fe³⁺) form, which impairs hemoglobin’s ability to bind and release oxygen effectively and shifts the oxygen dissociation curve to the left, resulting in tissue hypoxia despite a normal or elevated partial pressure of oxygen (PaO₂) [2]. This phenomenon often goes unrecognized because standard pulse oximetry cannot differentiate methemoglobin from oxyhemoglobin, frequently yielding falsely low or fixed oxygen saturation readings around 85% [6].

Acquired methemoglobinemia is most commonly triggered by exposure to oxidizing agents such as nitrates, sulfonamides, and topical anesthetics, especially benzocaine [4,6]. Benzocaine is widely used for procedures like upper endoscopy, bronchoscopy, and transesophageal echocardiography (TEE) and is well documented in association with methemoglobinemia [5]. Factors increasing susceptibility include high doses of benzocaine, repeated application, broken or inflamed mucosa, and underlying comorbidities such as renal or pulmonary disease [1,3].

Clinical manifestations vary based on methemoglobin (MetHb) levels and patient factors. Symptoms may range from cyanosis and fatigue to more serious complications such as seizures, arrhythmias, and coma at MetHb levels exceeding 30-50% [5,7]. However, the severity of symptoms does not always correlate linearly with MetHb levels, making clinical judgment essential. Diagnosis is confirmed with arterial blood gas and co-oximetry, which directly measure MetHb concentrations and reveal the characteristic “saturation gap” between pulse oximetry (SpO₂) and calculated arterial saturation (SaO₂) [6].

Treatment is generally recommended for symptomatic patients or when MetHb levels exceed 20-30%, although lower thresholds may warrant treatment in high-risk or symptomatic individuals. Methylene blue, administered at 1-2 mg/kg intravenously over five minutes, is the first-line antidote. It serves as an electron donor in the nicotinamide adenine dinucleotide phosphate (NADPH)-dependent MetHb reductase pathway, restoring hemoglobin to its functional ferrous state [7,8]. In patients with contraindications to methylene blue, such as those with G6PD deficiency or known hypersensitivity, alternative treatments include high-dose ascorbic acid, exchange transfusion, or hyperbaric oxygen in severe or refractory cases [5,6].

In our case, early recognition of the saturation gap, absence of overt pulmonary symptoms, and recent benzocaine exposure led to a prompt diagnosis and rapid clinical improvement following methylene blue administration. This case underscores the need for vigilance, especially in procedural settings where benzocaine is used. Preventive strategies should include minimizing benzocaine use in high-risk individuals and opting for safer alternatives such as lidocaine when appropriate [4].

This case aligns with previously reported literature [4,6,9] and reinforces the importance of timely identification and treatment of benzocaine-induced methemoglobinemia. The term “silent hypoxia” in this context refers to the absence of dyspnea or overt symptoms despite clinically significant hypoxemia. Recognition of this pattern allowed for early diagnosis and treatment, resulting in reversal of hypoxia and avoidance of unnecessary ICU escalation. Greater awareness of individual susceptibility, diagnostic pitfalls such as the saturation gap, and therapeutic considerations can improve patient safety in both inpatient and procedural settings. Furthermore, this case highlights an opportunity for institutions to reevaluate procedural protocols, particularly regarding the routine use of benzocaine, and consider safer alternatives like lidocaine in high-risk patients to enhance outcomes and reduce preventable complications.

## Conclusions

Benzocaine-induced methemoglobinemia is a serious yet reversible cause of sudden hypoxia, often overlooked due to its rare and silent presentation. This case underscores the importance of heightened clinical vigilance, particularly when topical anesthetics are used during diagnostic procedures. Recognizing methemoglobinemia based on subtle signs and initiating methylene blue promptly can dramatically improve outcomes. Increased awareness of this condition among proceduralists and critical care teams is essential for timely diagnosis and management.
